# A machine-learning-driven data labeling pipeline for scientific analysis in *MLExchange*

**DOI:** 10.1107/S1600576725002328

**Published:** 2025-05-12

**Authors:** Tanny Chavez, Zhuowen Zhao, Runbo Jiang, Wiebke Koepp, Dylan McReynolds, Petrus H. Zwart, Daniel B. Allan, Eliot H. Gann, Nicholas Schwarz, Daniela Ushizima, Edward S. Barnard, Apurva Mehta, Subramanian Sankaranarayanan, Alexander Hexemer

**Affiliations:** a Advanced Light Source, Lawrence Berkeley National Laboratory, Berkeley, CA 94720, USA; b Center for Advanced Mathematics for Energy Research Applications, Lawrence Berkeley National Laboratory, Berkeley, CA 94720, USA; c Molecular Biophysics and Integrated Bioimaging Division, Lawrence Berkeley National Laboratory, Berkeley, CA 94720, USA; d Berkeley Synchrotron Infrared Structural Biology Program, Lawrence Berkeley National Laboratory, Berkeley, CA 94720, USA; e National Synchrotron Light Source II Brookhaven National Laboratory,Upton NY 11973 USA; f Advanced Photon Source, Argonne National Laboratory, Lemont, IL 60439, USA; g Computational Research Division, Lawrence Berkeley National Laboratory, Berkeley, CA 94720, USA; h The Molecular Foundry, Lawrence Berkeley National Laboratory, Berkeley, CA 94720, USA; i Linac Coherent Light Source SLAC National Accelerator Laboratory,Menlo Park CA 94025 USA; j Center for Nanoscale Materials, Argonne National Laboratory, Lemont, IL 60439, USA; k Department of Mechanical and Industrial Engineering, University of Illinois Chicago, Chicago, IL 60607, USA; SLAC National Accelerator Laboratory, Menlo Park, USA

**Keywords:** X-ray scattering, data labeling, machine learning, user interfaces, feature extraction

## Abstract

This study introduces a novel labeling pipeline to accelerate the labeling process of scientific data sets by using artificial intelligence (AI)-guided tagging techniques. This pipeline includes a set of interconnected web-based graphical user interfaces (GUIs), where *Data Clinic* and *MLCoach* enable the preparation of machine learning (ML) models for data reduction and classification, respectively, while *Label Maker* is used for label assignment. Throughout this pipeline, data can be accessed through a direct connection to a file system or through *Tiled* for access through Hypertext Transfer Protocol (HTTP). Our experimental results present three use cases where this labeling pipeline has been instrumental for the study of large X-ray scattering data sets in the area of pattern recognition, the remote analysis of resonant soft X-ray scattering data and the fine-tuning process of foundation models. These use cases highlight the labeling capabilities of this pipeline, including the ability to label large data sets in a short period of time, to perform remote data analysis while minimizing data movement and to enhance the fine-tuning process of complex ML models with human involvement.

## Abstract 2

A web-based labeling pipeline is introduced that accelerates the annotation of large scientific data sets with artificial-intelligence-guided tagging techniques.

## 1. Introduction

The US Department of Energy (DOE) scientific user facilities (SUFs) have played an important role in scientific advancements and innovations by providing shared resources to scientists across a variety of research fields, such as materials science, physics, biosciences and others. With the mission of providing state-of-the-art research capabilities, many of these facilities have planned upgrades, including but not limited to increased brightness and coherence in synchrotron operations (Steier *et al.*, 2019 ▸; Collins *et al.*, 2017 ▸), increased power capability of accelerator-based neutron source operations (Champion *et al.*, 2017 ▸), and the creation of new computing capabilities such as the Integrated Research Infrastructure (IRI) vision (Miller *et al.*, 2023 ▸) and a High Performance Data Facility (HPDF) (Office of Science, 2023 ▸). There is an opportunity to exploit existing machine learning (ML) capabilities to enhance and accelerate the analysis of the large quantities of data collected at these facilities through the implementation and deployment of robust analysis pipelines, where users can make use of adaptable and scalable ML approaches through graphical user interfaces (GUIs). With such ML capabilities, users will have the ability to process their data while experiments are ongoing, and will potentially be able to use these results to push their operations further towards autonomous data collection procedures.

Nowadays, ML-based analysis techniques have obtained promising results across different applications at SUFs, such as autonomous experiments in electron and scanning probe microscopy (Kalinin *et al.*, 2021 ▸; Roccapriore *et al.*, 2022 ▸), data segmentation in X-ray tomography (Rippner *et al.*, 2022 ▸; Waldner *et al.*, 2024 ▸), phase identification in X-ray diffraction (Zhao *et al.*, 2023 ▸; Szymanski *et al.*, 2023 ▸), and pattern classification in X-ray scattering (Hadi Kiapour *et al.*, 2014 ▸). Previous work has highlighted the importance of employing labeled experimental data for training purposes to enhance the performance of ML models for micro-X-ray diffraction mapping and X-ray scattering pattern recognition with experimental data sets (Zhao *et al.*, 2023 ▸; Hadi Kiapour *et al.*, 2014 ▸). A limiting factor in adopting some of these existing ML capabilities in SUF operations is the availability of ground-truth information for training and quantitative evaluation purposes. This is due to the need for domain knowledge expertise and the significant amount of time required to annotate these data sets accurately. Therefore, there is an increasing need for a labeling pipeline specifically designed to accelerate the annotation process of intrinsic scientific data sets.

The literature presents studies that explore the implementation of GUIs for manual and semi-automated labeling techniques in image data sets, aiming to expedite this process. For instance, *DetEdit* (https://github.com/MarineBioAcousticsRC/DetEdit) is a MATLAB-based GUI for interactive visualization, exploration and annotation of acoustic data through the definition of labeling thresholds or manually assigned labels (Solsona-Berga *et al.*, 2020 ▸). An alternative annotation workflow, called *ilastik*, offers a *PyQT* GUI with flexible ML-based classifiers to accelerate the pixel-wise annotation process for segmentation, object detection and object tracking within multidimensional images (Berg *et al.*, 2019 ▸). Similarly, *Snorkel* (https://snorkel.ai) provides a Python software package compatible with *Jupyter* notebooks that presents a collection of labeling functions to be applied through programmatic labeling, where the estimated labels are parsed through a generative model as noisy ground truth to be ultimately tagged by a discriminator (Ratner *et al.*, 2020 ▸). While *Snorkel* offers a web-based ML operations (MLOps) interface with these capabilities, it is currently not open source and can only be accessed with a subscription. Musleh *et al.* (2023 ▸) introduced a systematic comparison of annotation tools for AI applications in ophthalmology, which identified a total of 131 annotation tools across the web. From that study, we can highlight two open-source web-based annotation tools: *makesense.ai* (Skalski, 2019 ▸) and *CVAT* (CVAT.ai Corporation, 2023 ▸), which have been extensively used in scientific applications. While both tools enable labeling of data sets both manually and through pre-trained object-detection ML models, they present limited capabilities to fine-tune models within the same pipeline. ML algorithms for object detection face further limitations in SUF applications, such as classifying structural patterns in X-ray scattering, where patterns may coincide or overlap with or without correlation (Huang *et al.*, 2021 ▸). Therefore, scientists at SUFs require an open-source web-based labeling pipeline that can easily adapt to large-scale data sets, with the option to train and fine-tune custom­izable ML models that can cater for different use cases, ideally within the same pipeline.

*MLExchange* is an open-source web-based MLOps platform that aims to close the gap in adopting ML-based solutions for scientific discovery. It is currently being developed in collaboration with scientists across six DOE-funded SUFs: the Advanced Light Source (ALS) at Lawrence Berkeley National Laboratory, the Advanced Photon Source and Center for Nanoscale Materials at Argonne National Laboratory, the Linac Coherent Light Source at SLAC National Accelerator Laboratory, the National Synchrotron Light Source II (NSLS-II) at Brookhaven National Laboratory (BNL), and the Center for Nanophase Materials Sciences at Oak Ridge National Laboratory (Zhao *et al.*, 2022 ▸). Within its ecosystem, users have access to an assortment of both traditional analytical algorithms and ML-based solutions for a diversity of scientific endeavors, such as peak detection in X-ray diffraction data, the detection of particle orientation in scanning electron microscopy data and the segmentation of three-dimensional tomographic data (Hao *et al.*, 2023 ▸). *MLExchange* empowers users to share assets, such as algorithms and GUIs, across its community so as to remain at the forefront of data analysis techniques. A previous report introduced the core services to enable the operation of *MLExchange*, while highlighting some of its existing capabilities in the realm of image annotation (Zhao *et al.*, 2022 ▸). Further expanding these efforts, this paper introduces a novel labeling pipeline that makes use of a variety of ML algorithms based on unsupervised and supervised learning approaches to accelerate the arduous task of labeling large scientific data sets using three web-based applications: *Label Maker*, *Data Clinic* and *MLCoach*.

*Label Maker* offers a web user interface where users can visualize and tag data sets through manual labeling or AI-guided techniques based on similarity-based querying and pre-trained discriminators. *Label Maker* connects with two other *MLExchange* applications, *Data Clinic* and *MLCoach*, where the ML models are trained prior to labeling. Ongoing development efforts are expanding *Label Maker*’s capabilities by adding a third web tool, *Latent Space Explorer*, for latent space visualization and data clustering. Users can access their data sets through a file system or through a data access service called *Tiled* (Rakitin *et al.*, 2022 ▸). To demonstrate the labeling capabilities of *Label Maker*, this study highlights three use cases where this pipeline has enabled labeling of large historical X-ray scattering data sets, the remote analysis of resonant soft X-ray scattering (RSoXS) data and the process of fine-tuning foundation models with human feedback.

This paper is organized as follows. Section 2 introduces the architecture and software components of the labeling pipeline, Section 3 describes the ML applications that support the operation of this pipeline, Section 4 presents the experimental results, Section 5 discusses the capabilities of this pipeline and outlines future development plans, and Section 6 summarizes the conclusions of this study.

## 2. Labeling data pipeline

**Figure 1:**
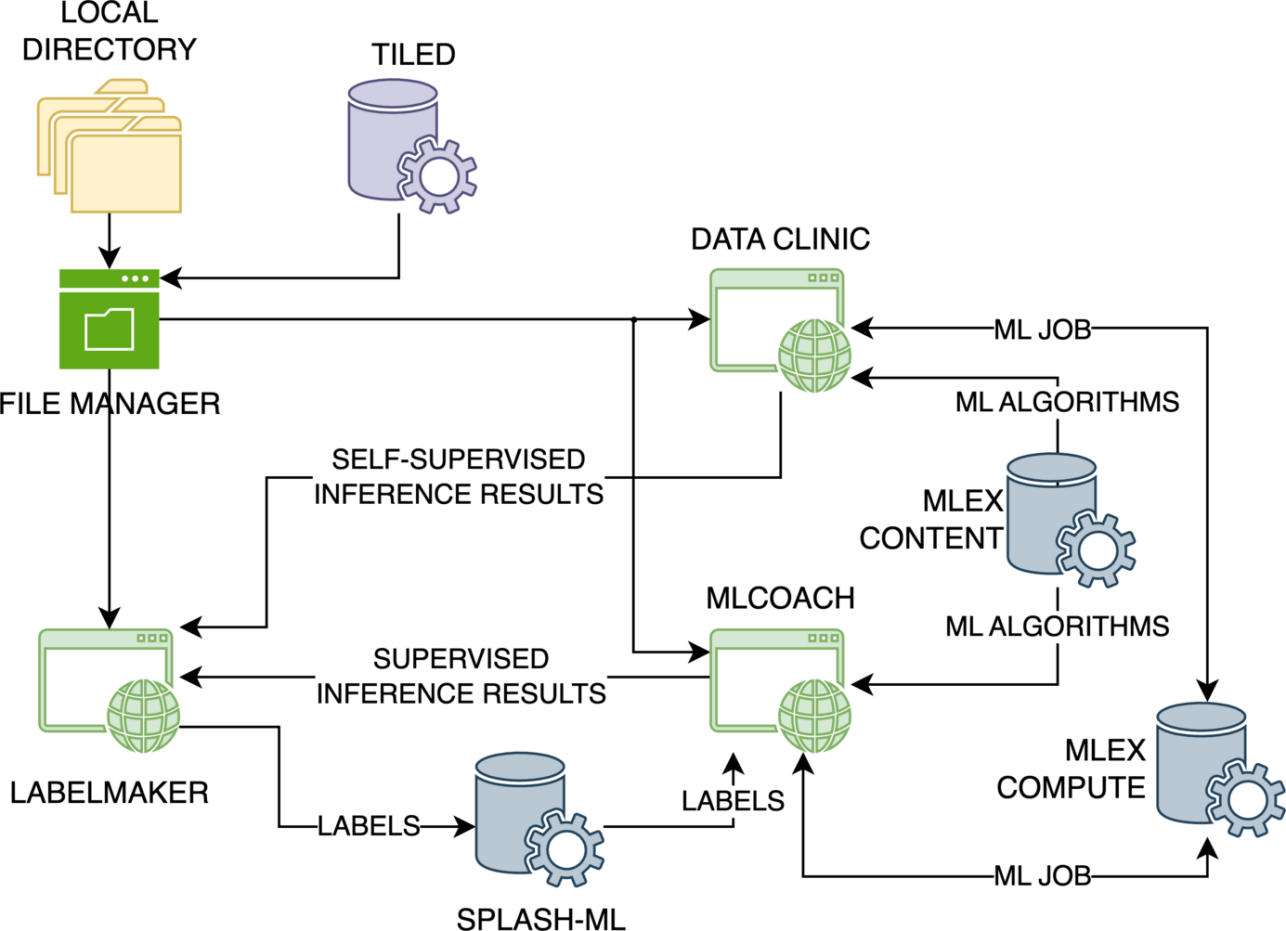
Software architecture diagram of the labeling pipeline, which consists of three front-end applications, *Label Maker*, *Data Clinic* and *MLCoach*, that are supported by four back-end services, *Tiled*, *MLExContent*, *MLExCompute* and *Splash ML*.

The proposed labeling pipeline consists of three front-end applications and four back-end services, as shown in Fig. 1 ▸. Among the front-end components, the pipeline strategically connects the image labeling interface (*Label Maker*) to two supporting applications for ML analysis (*Data Clinic* and *MLCoach*), such that their trained ML models can be used to accelerate the labeling process. All the web-based user interfaces in this pipeline were developed using *Dash* (Plotly Technologies Inc., 2015 ▸). Further details of the operation of these applications are given in the following section.

In the back-end, *Label Maker* makes use of two existing core application programming interface (API) components within the *MLExchange* platform, *MLExContent* and *MLExCompute*, that catalog ML algorithms within a registry and orchestrate the execution of ML workflows, respectively (Zhao *et al.*, 2022 ▸). For instance, *Data Clinic* and *MLCoach* make use of these two services to retrieve all the available ML techniques for their respective analyses and to supervise the execution of their training and inference routines, respectively. The two remaining services perform data management tasks, where *Tiled* enables fast data access via Hypertext Transfer Protocol (HTTP) and *Splash ML* bookkeeps the assigned labels within a database. A detailed description of these services is included below.

### 2.1. Data access

The labeling pipeline utilizes a custom *Dash* component called *File Manager* which enables users to access sets of images from a file system or from *Tiled* uniform resource identifiers (URIs). With this tool, users can load new data sets, bring back a previously loaded data set or clear the current data set of interest from a front-end interface. During data loading operations, users have the ability to select multiple file directories or tiled URIs to span their data set of interest across the pipeline. The applications in this labeling pipeline also offer a set of data transformations for both visualization and analysis purposes, such as log-transformation and percentile-based data normalization.

When loading data from a file system, *File Manager* walks the directory of interest and lists image files, such as PNG, JPEG/JPG and TIF/TIFF. This component also allows web applications to display the image of interest in full resolution or resize it, which is beneficial for the visualization of image galleries. Alternatively, *File Manager* makes use of *Tiled*, which is a data access service for data set types, ranging from data frames to image sets, with enforcement of access control. *Tiled* facilitates data search and structured chunkwise access to those data in a variety of formats that are compatible with data science packages in Python, regardless of their source format (Rakitin *et al.*, 2022 ▸). On top of providing data access, *Tiled* enables access to sections of the data set in original or reduced resolutions, which is particularly important for the operation of this labeling pipeline. The integration of *Tiled* within *Label Maker* enables users seamlessly to visualize, label and analyze remote data without transferring the complete data set to the pipeline’s location.

The labeling pipeline does not download the *Tiled* data set in most use cases, except for ML-based training purposes. Given that training data are conventionally accessed multiple times across the training iterations, the labeling pipeline downloads a copy of the training set from *Tiled* prior to its analysis. For inference purposes, *Tiled* data are not downloaded since data points are accessed a single time in this process. To support this operation, custom *PyTorch* and *Tensorflow* data sets have been created for the ML algorithms in this labeling pipeline.

The experimental results in this study employed a public *Tiled* server that can be accessed without an API key. While it is possible to connect to an API-key-protected *Tiled* server by manually fixing its access key within the labeling pipeline components, we are currently exploring improved and secure approaches to parse and dynamically update API keys among components without requiring users to re-authenticate at multiple stages of their session.

### 2.2. Label management

The labeling pipeline makes use of *Splash ML* as the bookkeeping service for labels, which consists of an API service and a database. To operate, this service stores the label information associated with a data set of interest within a database, where the data sets are identified according to a URI. The advantage of this service is its fast access to tags, thanks to its index-based search and the portability of the labeled information through the API service.

Within *Label Maker*, users can store a set of labels as a tagging event in *Splash ML*, where these events can be queried according to an assigned tagger ID and a creation timestamp. Hence, users can easily access previously assigned labels and modify them accordingly across the web interfaces within the labeling pipeline for version control purposes. Bookkeeping of tagging events becomes crucial for the correct operation of the supervised ML classifiers within this pipeline since they seamlessly retrieve the label information from *Splash ML*.

## 3. Front-end applications

This section further introduces the detailed operation of the front-end applications that support the proposed data analysis pipeline.

### 3.1. Data clinic

**Figure 2:**
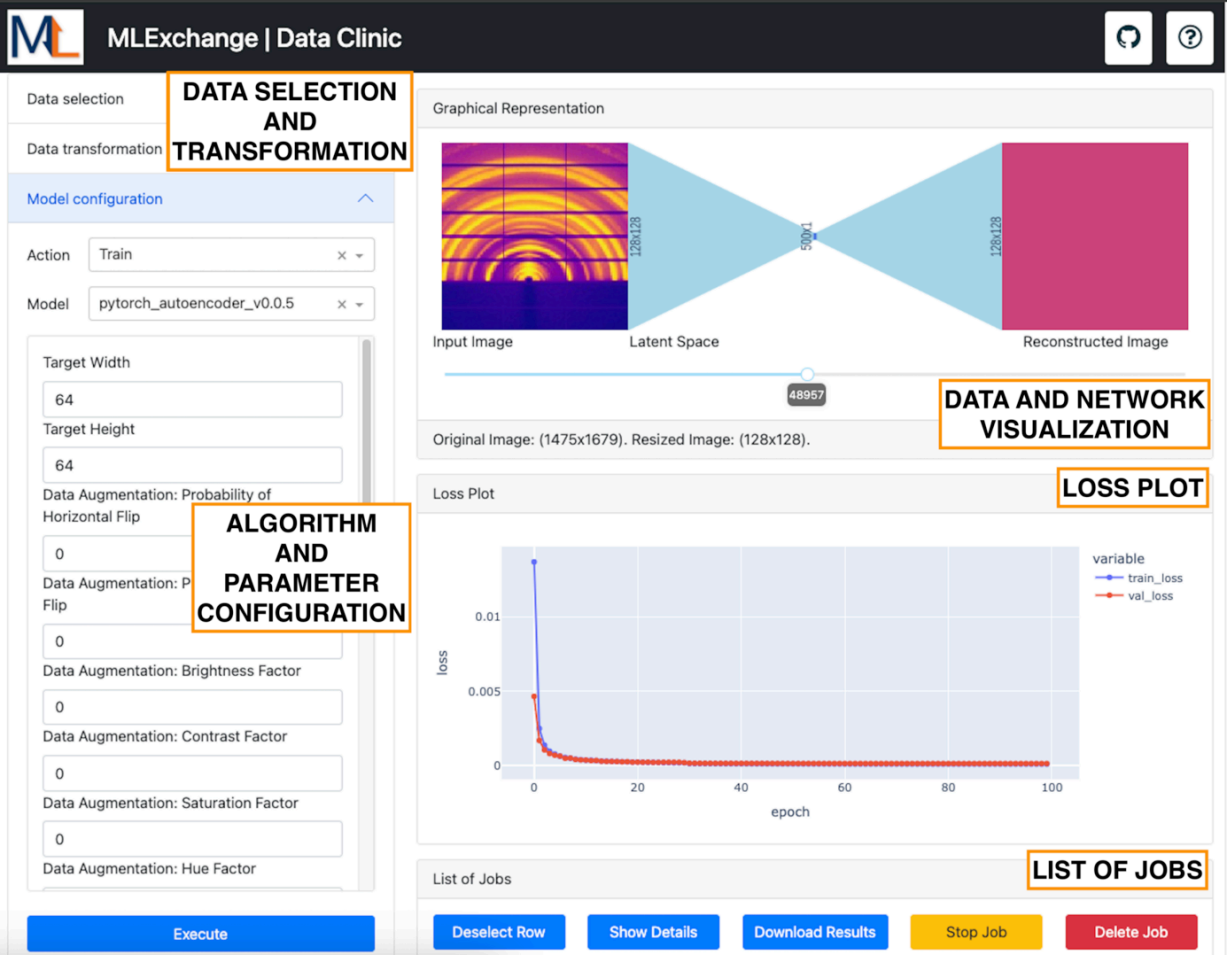
*Data Clinic* front-end interface. This application presents five main panels for data set selection and transformation, algorithm and parameter selection, graphical representation of the neural network, the loss plot associated with a selected training job, and a list of training and inference jobs.

*Data Clinic* is an application that enables latent space exploration of image data sets with customizable neural networks through self-supervised learning, and its front-end interface is shown in Fig. 2 ▸. Through this application, users can obtain a low-dimensional representation of their data set of interest, also referred to as latent or embedding space, where data points with similar characteristics are located in close proximity within the latent space (Xie *et al.*, 2009 ▸). To operate, this application retrieves compatible latent space extraction algorithms through *MLExContent*. When a user selects an algorithm from the dropdown menu, the parameter widget in the left-hand panel auto-populates using the information retrieved from *MLExContent* to adapt the GUI rapidly to the ML parameters of the selected algorithm. The graphical representation in the top right-hand panel dynamically displays the impact of modifying the resolution of the input image and the latent space dimension as a bottleneck in the neural network.

One of the main advantages of this web-based interface is that training and inference workflows are managed by the *MLExCompute* API in the *MLExchange* ecosystem, where these routines are executed in decoupled software containers running in the background. Therefore, new algorithms for latent space analysis can be easily integrated, making this application highly scalable. Additionally, users can check on the status of their running workflows at any point in time without the need to maintain an active session to the URL.

Overall, *Data Clinic* offers users a scalable web tool to analyze the impact of different latent space sizes based on the data reconstruction performance of the selected ML algorithm. Further details on the data processing steps across this interface are described as follows.

#### 3.1.1. Tunable autoencoders

**Figure 3:**
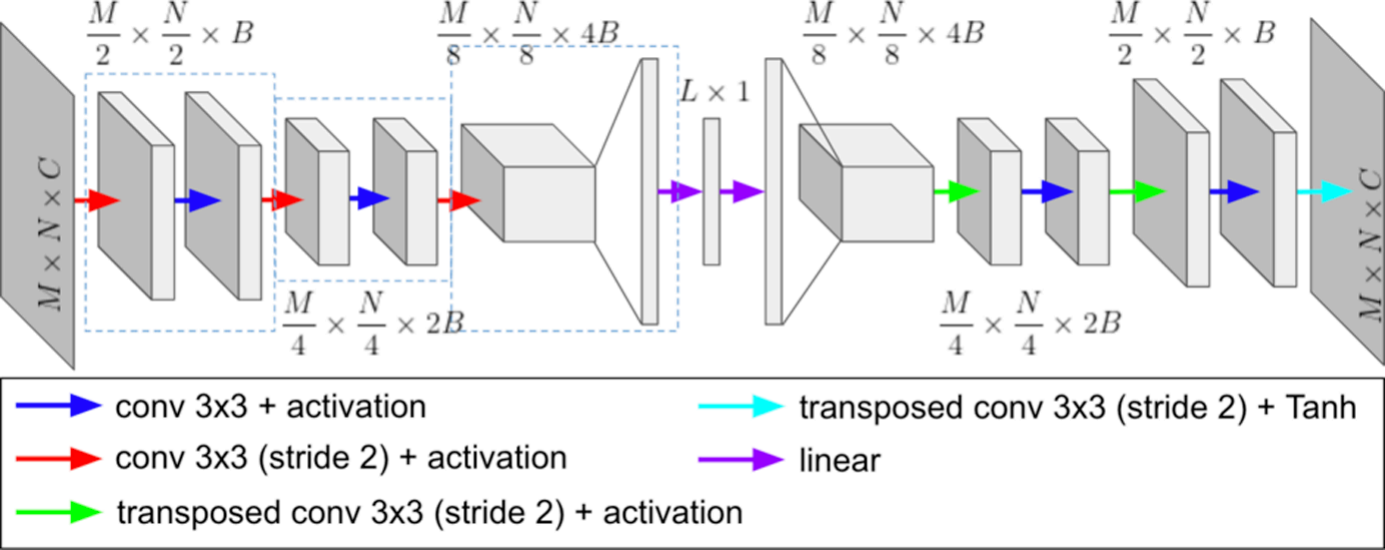
Architecture of a convolutional autoencoder of depth 3, where 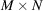 corresponds to the size of the input image, *B* represents the number of base channels and *L* is the latent space size.

Currently, the latent space extraction algorithm in *Data Clinic* corresponds to a convolutional autoencoder with tunable architecture parameters, similar to that reported by Lippe (2023 ▸). An autoencoder is a type of neural network that encodes a data set of interest into a low-dimensional vector and uses this representation to reconstruct (decode) the original input. Autoencoders are commonly used for data compression purposes and feature extraction (Rumelhart *et al.*, 1986 ▸; Hinton & Salakhutdinov, 2006 ▸; Meng *et al.*, 2017 ▸). A convolutional autoencoder uses convolutional and pooling layers for feature extraction, which provides a more localized embedding learning approach (Chavez *et al.*, 2022 ▸). Among the customizable network parameters offered in *Data Clinic*, we can highlight base channel size, latent dimension size and network depth. A diagram of a tunable network of depth 3 is shown in Fig 3 ▸, where 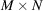 corresponds to the size of the input image, *B* represents the number of base channels and *L* is the latent space size. Note that, even though the proposed autoencoder can adapt to a wide selection of parameters, there may exist some combinations that span an unfeasible architecture. For instance, the selection of the network depth *d* is limited by the size of the input image, since the size of the layer prior to flattening is 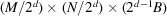, where 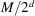 and 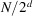 should be greater than 1. Hence, it is crucial for users to select carefully a suitable combination of parameters for their data set of interest.

To reinforce further the estimation of the latent features, this algorithm offers data augmentation options to be randomly applied solely to the input images per batch for augmentation-invariant models, or to both input and output images per batch for other use cases. These augmentation tools include random horizontal and vertical flips, and random changes to brightness, contrast, saturation and hue levels. Users have the option to set up a random seed to ensure reproducible augmentation results. In preparation for the training step, batches of images are retrieved from a directory or *Tiled* to be resized later according to a user-defined target dimension, which can accommodate the analysis of large data sets.

For training purposes, this algorithm offers a wide selection of optimizers and criteria with a customizable learning rate and number of epochs. At the end of every epoch, the loss plot in the front-end interface is updated with both train and validation losses in real time until the training routine is completed. While the loss plot already provides an evaluation metric of the performance of the trained network, users can choose to perform inference on a given data set to provide a visual inspection of the quality of the reconstructed images at the output of the autoencoder. In addition, the output of the inference analysis provides the estimated latent vectors for all the elements of the data set to be used later by *Label Maker* for similarity-based queries.

### 3.2. MLCoach

**Figure 4:**
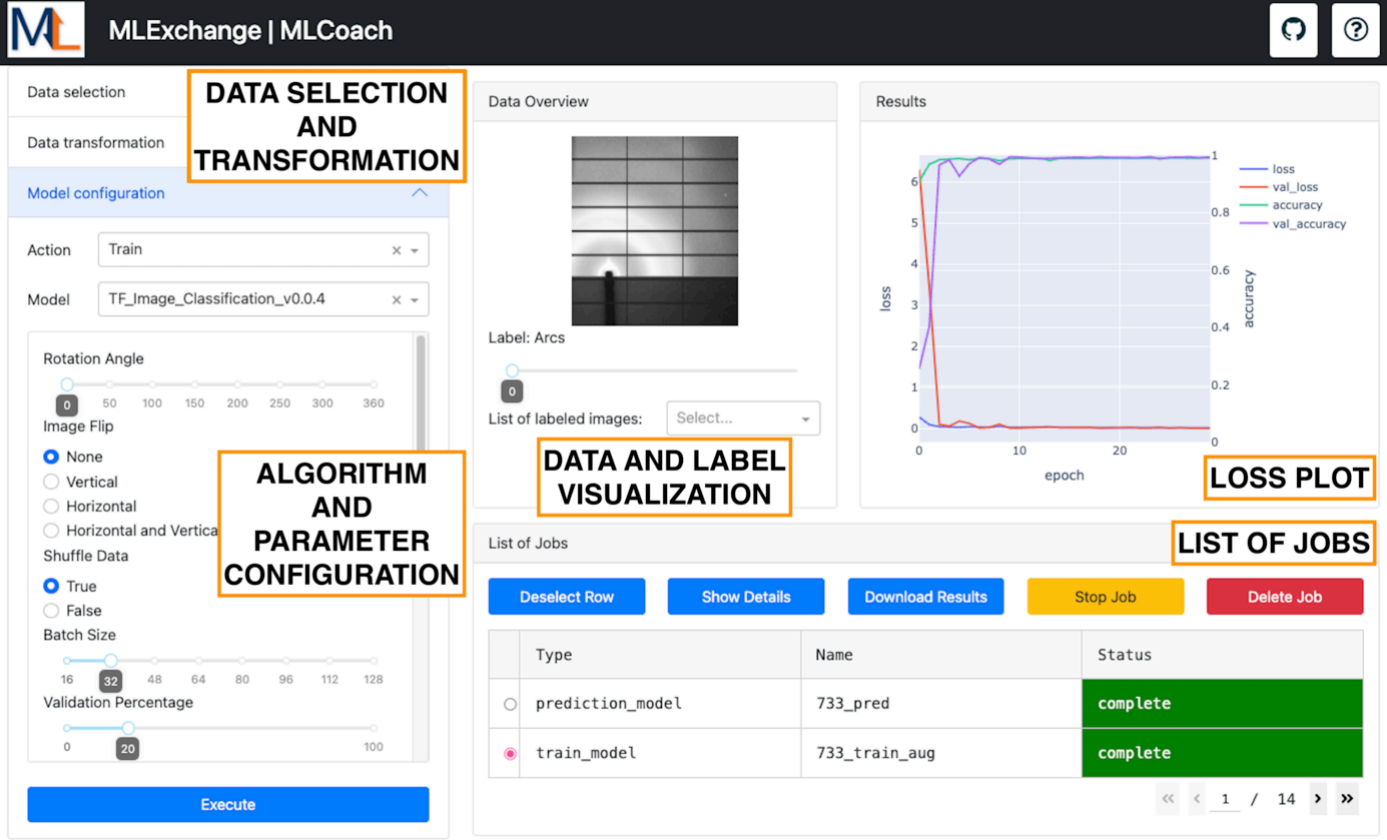
*MLCoach* front-end interface. This application presents five main panels for data set selection and transformation, algorithm and parameter selection, data and label visualization, the loss plot associated with a selected training job, and a list of training and inference jobs.

*MLCoach* is an application for image classification purposes, as shown in Fig. 4 ▸. Similarly to *Data Clinic*, this interface also retrieves compatible algorithms and runs workflows through the *MLExContent* and *MLExCompute* services in *MLExchange*. Hence, the user’s interaction with respect to selecting an algorithm, setting parameters and visualizing real-time training metrics is very similar to that described before. However, the key difference between these applications is the fact that *MLCoach* makes use of supervised learning algorithms, which ultimately requires a labeled input data set for training.

In general, *MLCoach* retrieves the labels associated with a data set of interest through a tagging event in *Splash ML*, where the label information is collected from a database. Once the data set is defined, the labeled information is displayed in the graphical representation panel of this web interface as a point of reference. Further details about the ML algorithms available in this application are introduced as follows.

#### 3.2.1. Probabilistic classifiers

*MLCoach* currently offers a wide variety of deep-learning architectures for image classification purposes, such as Xception (Chollet, 2017 ▸), Visual Geometry Group (VGG) (Simonyan & Zisserman, 2014 ▸), Residual Network (ResNet) (He *et al.*, 2016 ▸), Inception (Szegedy *et al.*, 2016*b* ▸), Dense Convolutional Network (DenseNet) (Huang *et al.*, 2017 ▸), InceptionResNet (Szegedy *et al.*, 2016*a* ▸) and Neural Architecture Search Networks (NASNet) (Zoph *et al.*, 2018 ▸). Users are given the option to train these architectures from scratch or make use of pre-trained weights, *e.g.* ImageNet, to start the training process. ImageNet is a large training set that contains more than 14 million labeled images (Deng *et al.*, 2009 ▸), from which subsets of approximately 1.4 million and 1000 classes have been used to pre-train these *Tensorflow*-based networks (Abadi *et al.*, 2015 ▸). Regardless of the selected architecture, the proposed probabilistic classifier employs a softmax activation layer to estimate the label probability per image at the output of the neural network. For reasons of architecture compatibility, all the input images are transformed to RGB color space and resized to 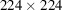 for VGG, ResNet and DenseNet, 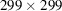 for Xception, Inception and InceptionResNet, and 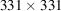 for NasNets.

Similarly to *Data Clinic*, data augmentation options are available in *MLCoach*, such as random image rotation and flips, where a random seed can be pre-defined for data reproducibility. While setting up the training routine, users can also select the loss function, optimizer, batch size and number of epochs to customize their analysis. The training metrics are also displayed in real time in terms of loss and accuracy per epoch for both training and validation sets.

During the inference routine in *MLCoach*, both labeled and unlabeled data sets are accepted within the interface. A summary of the resulting probabilities per image is displayed in the top right-hand panel of the web interface as a bar plot, where labels are color coded for easy visual inspection. The overall classification results and the supervised feature vectors from the second to the last layer are stored in data frames to be used later by *Label Maker*.

### 3.3. Label Maker

**Figure 5:**
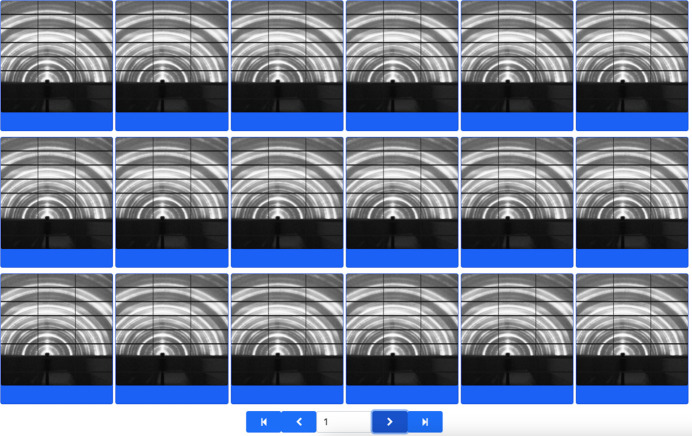
*Label Maker* similarity-based labeling approach, where feature vectors from *Data Clinic* and *MLCoach* are used to estimate neighbor ranking among data points in the data set of interest. Using this approach, it is possible to label batches of similar images simultaneously.

*Label Maker* is the core application of the labeling pipeline, where labels are assigned, modified, deleted and stored. To operate, this interface displays the data set of interest in the right-hand column for visual inspection, where a customizable number of images are loaded per page as shown in Fig. 5 ▸. In the left-hand panel, users can add new labels, modify the label colors or delete the labels if necessary. The top row of this section indicates the labeling approaches that are available within this application, which are manual and two AI-guided labeling approaches through similarity-based queries and probability-based label assignment.

#### 3.3.1. Manual labeling

Under the manual operation mode, users can assign labels to a single image or group of images simultaneously by clicking the elements to be tagged, followed by their corresponding label. *Label Maker* tracks the label assignment process by color coding each image within the data set according to the user’s selection. Users can easily check their labeling progress through the progress bar in the left-hand panel of the web browser, which graphically depicts the number of labeled images per class. To facilitate the labeling process, the interface allows users to use key bindings to assign labels, sort tagged images according to their label, hide labeled images and unlabel images as needed. Current labels can be stored in a database through *Splash ML*, where they can be easily retrieved at a later point in time. Alternatively, users can choose to download their labeling results as a table or as a ZIP file where the labeled images are organized into folders, with each folder corresponding to the label that was assigned in *Label Maker*. Such a directory structure is compatible with existing ML frameworks for easy categorization during their training process.

#### 3.3.2. Similarity-based labeling

To accelerate the labeling process further, *Label Maker* can make use of previously trained *Data Clinic* and *MLCoach* models to enable a similarity-based batch labeling process as represented in Fig. 5 ▸. The similarity metric used in this approach corresponds to the cosine distance between feature vectors, which are retrieved during the inference step. For the tunable autoencoders, the feature vectors correspond to the latent space representation of the input data at the bottleneck of the network, while for the probabilistic classifiers these vectors correspond to the second to last layer in their networks’ architectures. Thus, given the inference results of a *Data Clinic* or *MLCoach* model and an image of interest, all the elements within the data set are sorted such that the most similar image is located at the beginning of the sequence and the least similar image is at the end of the sequence. Once arranged, the user can proceed to label batches of similar images with a single label selection.

Considering that the presented autoencoders do not require labeled information for their corresponding training routines, this AI-guided labeling method with *Data Clinic* models is suitable for starting the tagging process in a completely unlabeled data set. Alternatively, users can make use of pre-trained *MLCoach* models to perform similarity-based querying through supervised learning approaches, analogous to *PyCBIR* (Araujo *et al.*, 2018 ▸).

#### 3.3.3. Probability-based labeling

**Figure 6:**
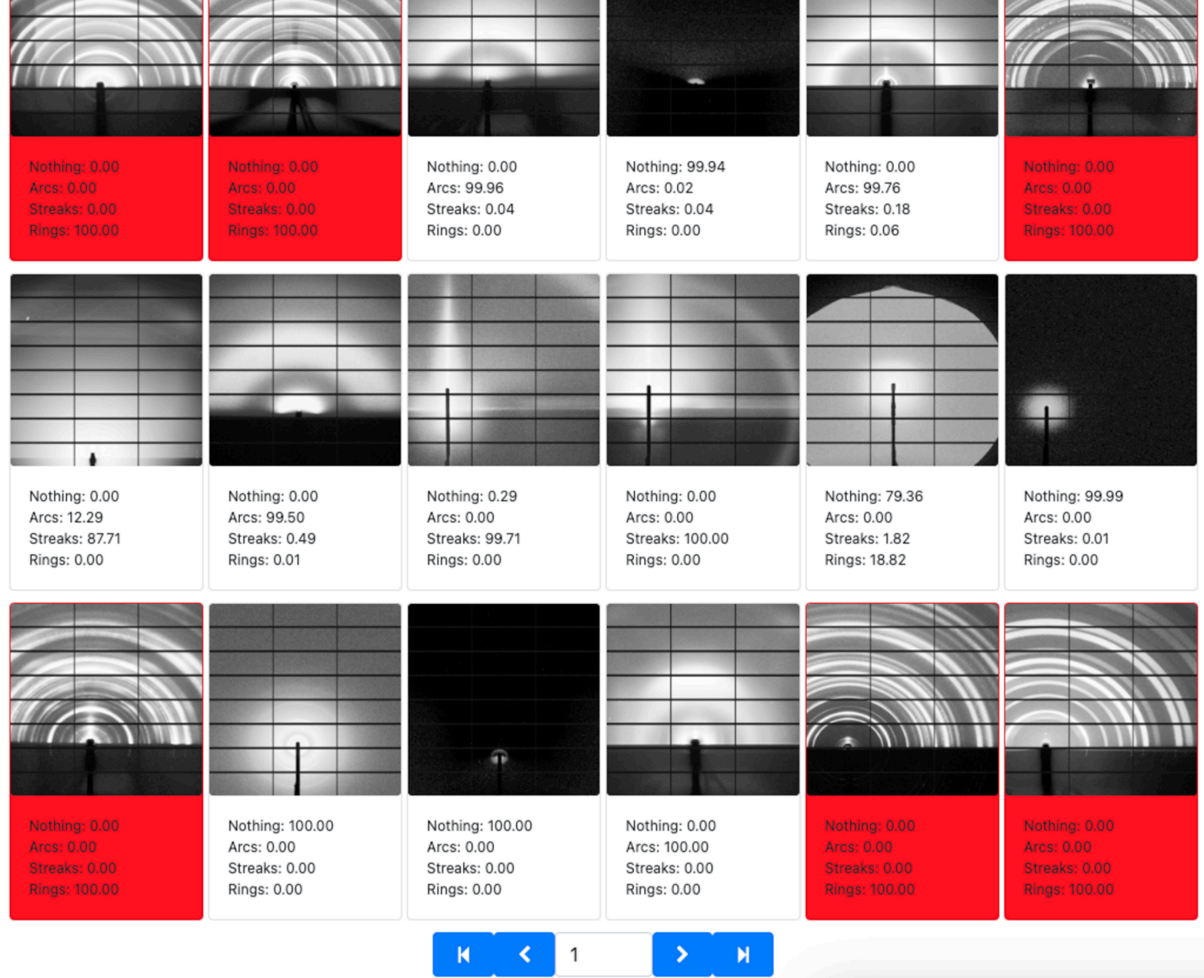
*Label Maker* probability-based label assignment, where classification results from previously trained *MLCoach* classifiers are used to automatically label a set of images that comply with a threshold condition defined by the user.

Alternatively, *Label Maker* can automatically tag unlabeled images within the data set of interest through the definition of a probability threshold. Given the inference results of a supervised probabilistic classification model trained in *MLCoach* and a user-defined probability threshold for a given label, every unlabeled image that presents a label probability equal to or higher than this threshold is automatically tagged under this operation mode (Fig. 6 ▸).

The selection of a suitable classification model is fundamental for the correct assignment of labels in this approach. Therefore, this mode of operation is suggested only after a significant portion of the data set of interest has already been labeled manually or through similarity-based approaches.

### 3.4. Latent Space Explorer

**Figure 7:**
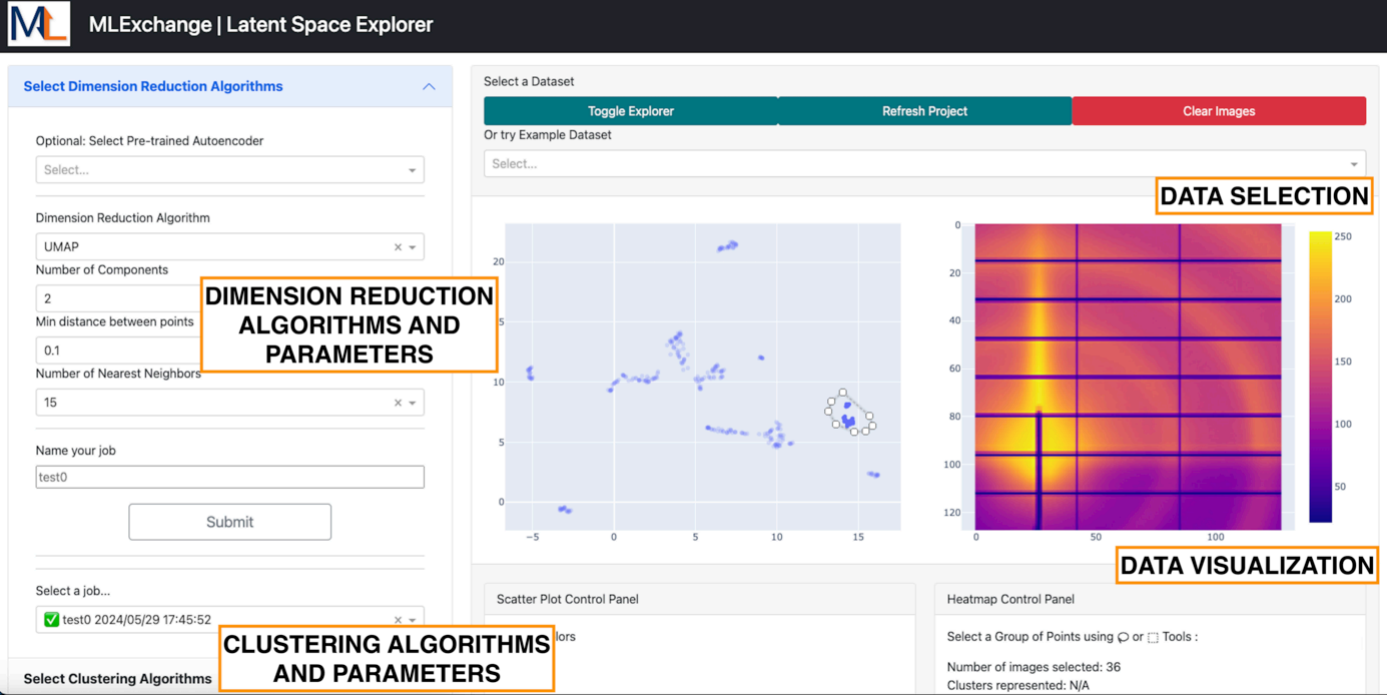
*Latent Space Explorer* front-end interface. This application presents four main panels for data selection, dimension reduction algorithm and parameter selection, clustering algorithm and parameter selection, and data visualization.

Within the *Label Maker* ecosystem, we are currently integrating an additional application called *Latent Space Explorer* for the visualization of latent space exploration. *Latent Space Explorer* is a web-based tool designed for exploring high-dimensional data through dimensionality reduction and clustering techniques, as shown in Fig. 7 ▸. Thanks to its intuitive interface, users can effortlessly navigate through data sets using *File Manager*’s data access capabilities. Central to its functionality is support for dimensionality reduction algorithms, including principal component analysi (PCA) (Abdi & Williams, 2010 ▸) and uniform manifold approximation and projection (UMAP) (McInnes *et al.*, 2018 ▸), empowering users to tailor the analysis to their specific needs by fine-tuning algorithm parameters through drop-down menus. The application also offers clustering capabilities, seamlessly integrating algorithms such as KMeans (Arthur & Vassilvitskii, 2007 ▸), density-based spatial clustering of applications with noise (DBSCAN) (Ester *et al.*, 1996 ▸) and hierarchical density-based spatial clustering of applications with noise (HDBSCAN) (McInnes *et al.*, 2017 ▸). This enables users to discern underlying patterns and structures within the data.

The application also provides interactive visualizations, including scatter plots of the reduced-dimensional data and informative heat maps of selected data points, where users can choose either the mean or standard deviation of the selected points of interest. In addition, statistical information is provided about selected data points, including the number of images selected, represented clusters and represented labels. *Latent Space Explorer* can also make use of pre-trained models from *Data Clinic* and *MLCoach*, allowing users to analyze pre-existing latent vectors for posterior analysis.

## 4. Experimental results

This section summarizes the experimental results obtained from the data analysis capabilities offered within this labeling pipeline. For this purpose, we introduce three real-world use cases where the labeling pipeline has, respectively, enabled the assignment of labels to large X-ray scattering data sets, facilitated the remote analysis of RSoXS data, and enhanced the fine-tuning process of ML foundation models for the gen­eration of X-ray scattering data sets. Additional information on data and code availability, as well as hyperparameter selections, is provided in Appendices *A*[App appa]–*C*[App appb][App appc].

### 4.1. Labeling large X-ray scattering data sets

Access to labeled scientific data sets is imperative for the development of robust trained ML models that can cater for the increasing data processing needs within DOE user facilities. For instance, the deployment of *Label Maker* has introduced a streamlined method to process large X-ray scattering data sets for pattern-recognition purposes. To showcase this capability, we have labeled approximately 80000 scattering images from an assortment of user experiments, including transmission and grazing-incidence scattering, collected on the SAXS/WAXS beamline 7.3.3 (Hexemer *et al.*, 2010 ▸) at the ALS over the course of ten years.

**Figure 8:**
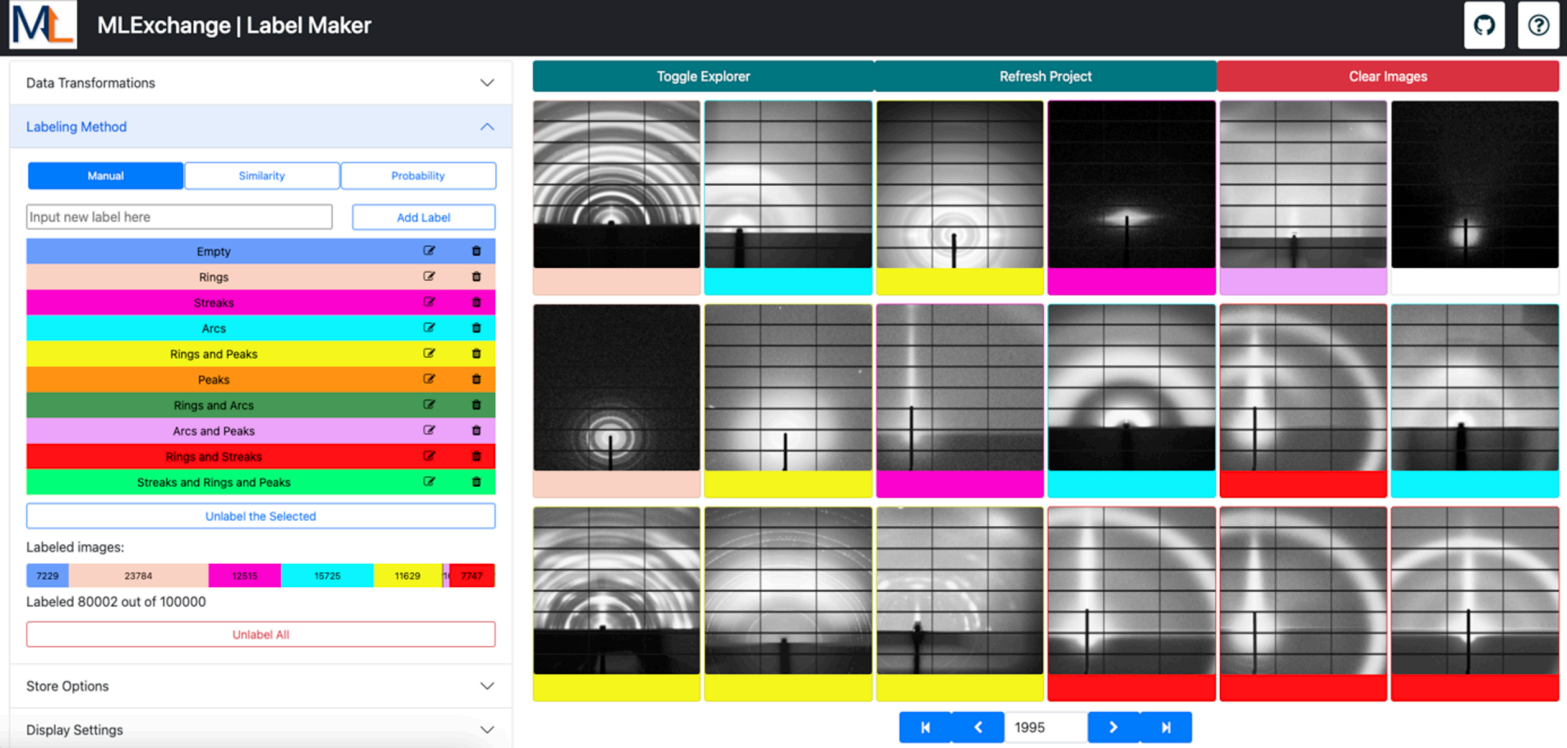
Screenshot of labeling progress, where 80000 images were labeled by a single user over the course of multiple non-consecutive labeling sessions. Labels were assigned according to the user’s defined color coding, where unlabeled images are presented in white, arcs in light blue, rings in light pink, streaks in bright pink, arcs and peaks in yellow, rings and streaks in red, and arcs and peaks in purple.

As shown in Fig. 8 ▸, we have identified ten classes within this data set, corresponding to different structural properties of the characterized materials, such as 16888 arcs, 7229 empty, 12840 peaks, 43437 rings, 162 rings and arcs, 11744 rings and peaks, 20377 streaks, 1001 arcs and peaks, 7747 rings and streaks, and 115 streaks, rings and peaks. The data set was labeled using a combination of manual, similarity-based and probability-based labeling within the *Label Maker* ecosystem. For the similarity-based instance, a tunable autoencoder was trained in *Data Clinic* with input size 128 × 128, base channel size 64, latent space dimension 500 and depth 4. The data set of interest was split for training and validation, where 20% was allocated to the validation set. The model was trained over 100 epochs with a batch size of 256, utilizing an initial learning rate of 0.001. The criterion was the minimum squared error loss, implemented with an ADAM optimizer (Kingma & Ba, 2017 ▸), with a learning rate scheduler that reduced the learning rate by a factor of 0.1 every 30 steps.

After labeling a total of 12257 images using the similarity-based approach, we determined that we had a significant amount of labeled data to train a ResNet classifier in *MLCoach* for further probability-based labeling. To train a robust model, the classifier was trained with data augmentation, including rotation angles up to 100° and random horizontal and vertical flips. We employed a batch size of 64, a validation split of 20%, a categorical cross-entropy loss function with an ADAM optimizer and a learning rate of 0.0001 for a total of 30 epochs. Upon completion of the training process, the classifier achieved accuracies of 0.99 for both the training and validation sets. All networks were trained on a single Nvidia GeForce RTX 4090 GPU with 23 GB capacity and an AMD Threadripper Pro 5965WX processor.

Fig. 8 ▸ displays a screenshot of the labeling interface, where we can observe the data set of interest with a total of 100000 images. On the left-hand side, a set of controls indicates live progress in the labeling task, with 80000 images labeled out of the total of 100000 images. A single user labeled this data set at a normal pace over multiple sessions, using a combination of manual and AI-guided labeling techniques for label assignment and review. As a reference, this user labeled 6588 images in a single labeling session of 42 min. If manually labeling these images without AI-guided techniques, a user would need to maintain an approximate manual labeling rate of 2.6 labels per second, with no pauses, to label the same number of images in the same time frame.

By comparison, alternative open-source labeling tools, such as *makesense.ai*, have notable differences to the labeling pipeline offered by *MLExchange*. For instance, *makesense.ai* allows users to run AI models locally [YOLOv5 (Jocher, 2020 ▸), COCO SSD (Liu *et al.*, 2015 ▸, 2014 ▸) and PoseNet (Kendall *et al.*, 2015 ▸)] or connect to AI servers through *Roboflow*’s (Dwyer *et al.*, 2024 ▸) inference servers, which is particularly powerful for production-ready ML models. While users can employ notebook interfaces to train their ML models for use with *makesense.ai*, there is an initial barrier to entry, as ML knowledge is required for the effective design and implementation of these algorithms. This challenge becomes even more pronounced in the context of SFUs, where samples, instrumentation, experimental setups and scientific drivers can vary significantly across experiments. To address this need, *MLExchange* provides a user-friendly framework that enables researchers to train models through intuitive web applications, such as *Data Clinic* and *MLCoach*, ensuring that their ML solutions align with their specific scientific goals.

**Figure 9:**
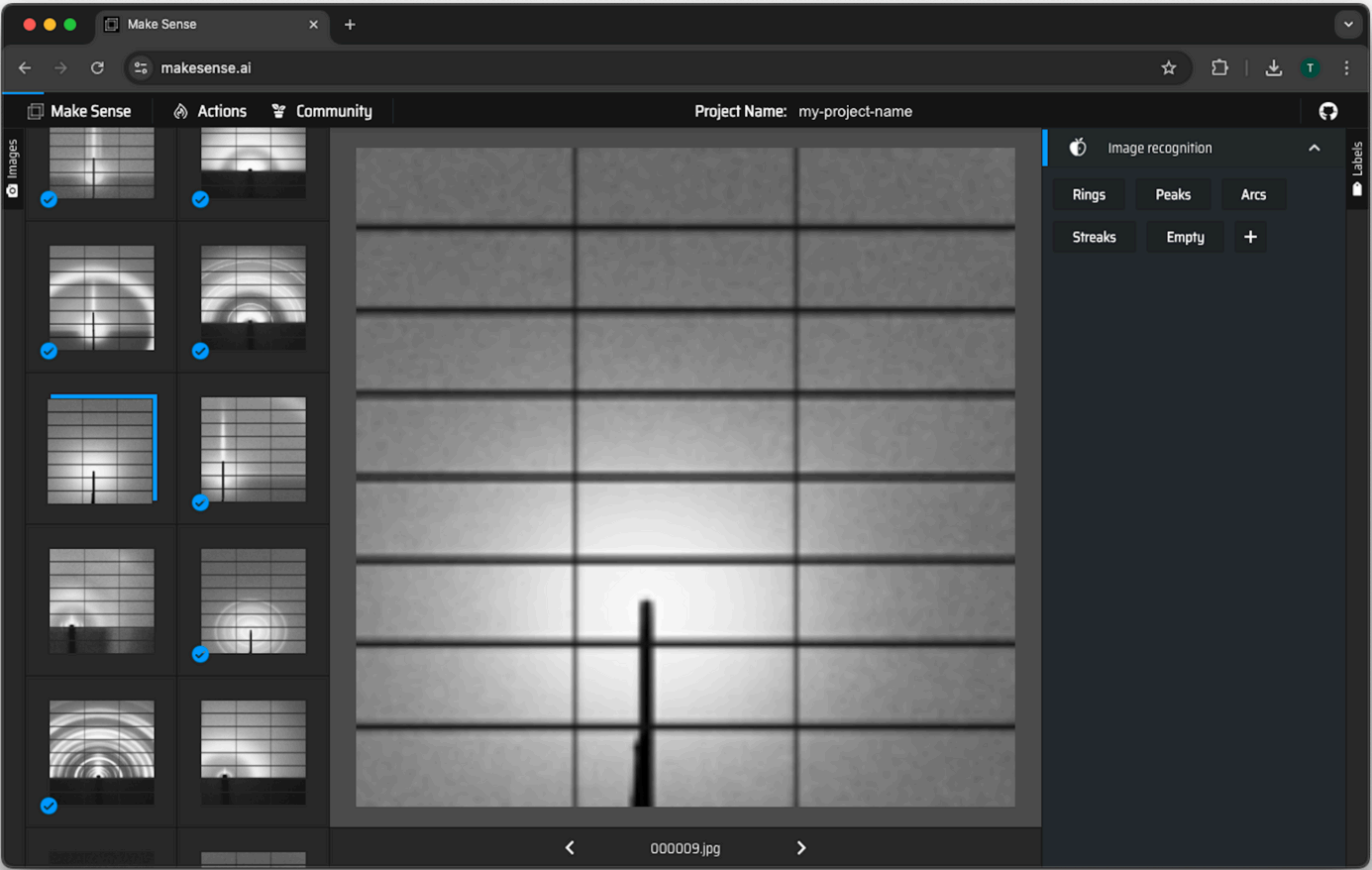
Web interface of *makesense.ai* (Skalski, 2019 ▸) with a subset of 400 images within the large X-ray scattering data set collected on beamline 7.3.3 at the ALS (Hexemer *et al.*, 2010 ▸). The left-hand sidebar displays a set of thumbnails from the data set, while the center of the interface provides a full-screen view of the selected thumbnail image. The right-hand sidebar includes the label options available for assignment to the selected image. Its navigation bar offers a set of actions from which we can highlight (i) *Run AI Locally*, allowing users to upload a locally trained model for object detection purposes, with support for models such as *YOLOv5* (Jocher, 2020 ▸), *COCO SSD* (Liu *et al.*, 2015 ▸; Lin *et al.*, 2014 ▸) and *PoseNet* (Kendall *et al.*, 2015 ▸); and (ii) *Connect AI Server*, enabling users to run a remote model by connecting to a specified *Roboflow* (Dwyer *et al.*, 2024 ▸) inference endpoint.

In addition, *makesense.ai* lacks tools for adjusting the brightness and contrast of images, making it challenging to determine whether an image represents background or contains low-intensity features (*e.g.* rings, peaks *etc.*). In contrast, *Label Maker* integrates percentile normalization and log transformations directly into its interface, facilitating the identification of these features. *makesense.ai* provides an intuitive interface that supports assigning multiple labels per image. However, its thumbnails only indicate whether an image has been labeled, without displaying the specific labels or the total number of images assigned to each label, as shown in Fig. 9 ▸. *makesense.ai* offers object detection assistance by suggesting labels within an image of interest, but it does not support batch labeling or similarity-based annotations, which are critical capabilities for classification workflows in *ML­Exchange*.

In terms of speed, *makesense.ai*’s manual labeling tool is comparable to *MLExchange* when labeling images one by one as their labeling features are very similar. Unlike the labeling pipeline in *MLExchange*, *makesense.ai* does not support the pre-selection of images for batch labeling or a similarity-based annotation suggestion. Without similarity-based label assignment, users need to label an assortment of images manually in order to train or fine-tune ML models on their own to be used in *makesense.ai*, which will require more time overall as labels will need to be assigned one by one. *MLExchange*’s support for *Tiled* facilitates data access and enhances its integration with existing pipelines for real-time analysis during experiments at SFUs.

### 4.2. Remote data analysis of RSoXS data

An alternative use case for *Label Maker* is enabling cross-facility remote data analysis through ML techniques. To demonstrate this, we used an existing RSoXS data set publicly available at https://tiled-demo.blueskyproject.io, which is hosted on the Amazon Web Services (AWS) cloud platform. This data set was collected on the RSoXS beamline at NSLS-II at BNL during alignment operations using *Bluesky* acquisition plans (Allan *et al.*, 2019 ▸). We selected 85 *Bluesky* runs with a total of 2697 RSoXS images of 1026 × 1024 pixels to be labeled using *Label Maker*.

**Figure 10:**
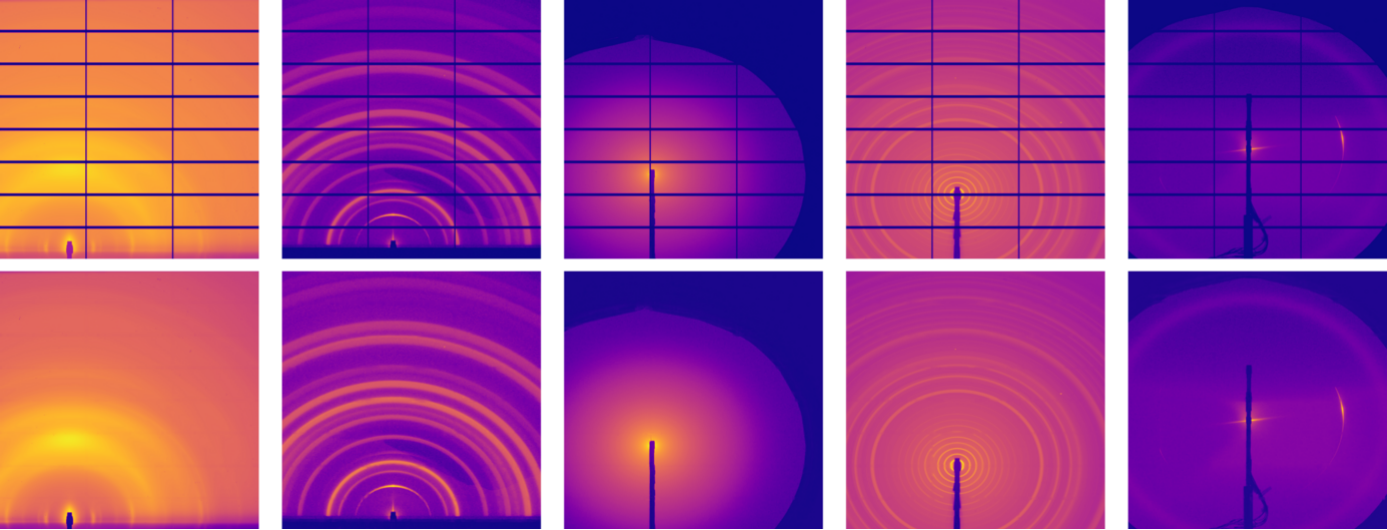
Sample images taken from the inpainted data set with 31252 X-ray scattering images. The top row illustrates the original masked experimental data, exhibiting missing pixel information located at the inter-module gaps, consistent with a PILATUS3 2M detector. The bottom row represents the inpainted images using a pre-trained MSDNet.

To avoid transferring the complete data set towards the pipeline’s computing location, we pre-trained a tunable autoencoder using 31252 inpainted X-ray scattering images (Fig. 10 ▸). This initial training set was acquired by randomly selecting a subset of user data from a series of transmission and grazing-incidence X-ray scattering user experiments on the ALS SAXS/WAXS beamline 7.3.3 (Hexemer *et al.*, 2010 ▸). The training set was inpainted prior to analysis to reconstruct the missing information located at the detector inter-module gaps in order to enhance the model transferability to the RSoXS data, which do not present these gaps. To achieve this, we made use of a pre-trained mixed-scale dense network (MSDNet) which is accessible at https://huggingface.co/phzwart/dlsia_inpainting_saxs_gisaxs (Chavez *et al.*, 2022 ▸; Roberts *et al.*, 2024 ▸).

The architecture of the pre-trained tunable autoencoder was defined using two different sets of parameters. The first architecture utilized an input size of 64 × 64 pixels, a base channel size of 32, depth 3 and a latent dimension size of 300. In contrast, the second architecture employed an input size of 256 × 256, a base channel size of 8, depth 5 and a latent dimension of 300. The training parameters corresponded to a learning rate of 0.001, batch size of 64, total number of epochs of 300 and learning rate decay of 0.1 every 100 epochs. The ADAM optimizer was used for the training process, which minimized the mean squared error as the evaluation metric (Kingma & Ba, 2017 ▸).

To enhance further the feature detection step of RSoXS data, we utilized a fraction of the *Tiled* data set (166 images) for fine-tuning purposes. Hence, we fine-tuned the previous network architecture with the same training parameters for another 200 epochs with a learning rate decay of 0.1 every 100 epochs. The networks were trained on a single Nvidia GeForce RTX 4090 GPU with 23 GB capacity and an AMD Thread­ripper Pro 5965WX processor. With this fine-tuned model, we inferred the feature vectors of the remaining data points in the set without further data transfers using custom *Tiled* data sets in *PyTorch*.

A log-transform followed by a percentile-based normalization step was applied to the RSoXS data set prior to fine-tuning to improve the visibility of the patterns presented within the image, which were otherwise not discernible through visual inspection. The log-transform applied was 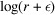, where *r* represents the pixel values of the min–max scaled image and ε = 10^−12^. We then proceeded to run a percentile-based normalization, using the first and 99th percentiles as boundaries, where the pixel intensities of the image of interest were set in the range [0, 1] with sharp streak features, similar to the inpainted images that were used to pre-train the tunable autoencoder in *Data Clinic*.

**Figure 11:**
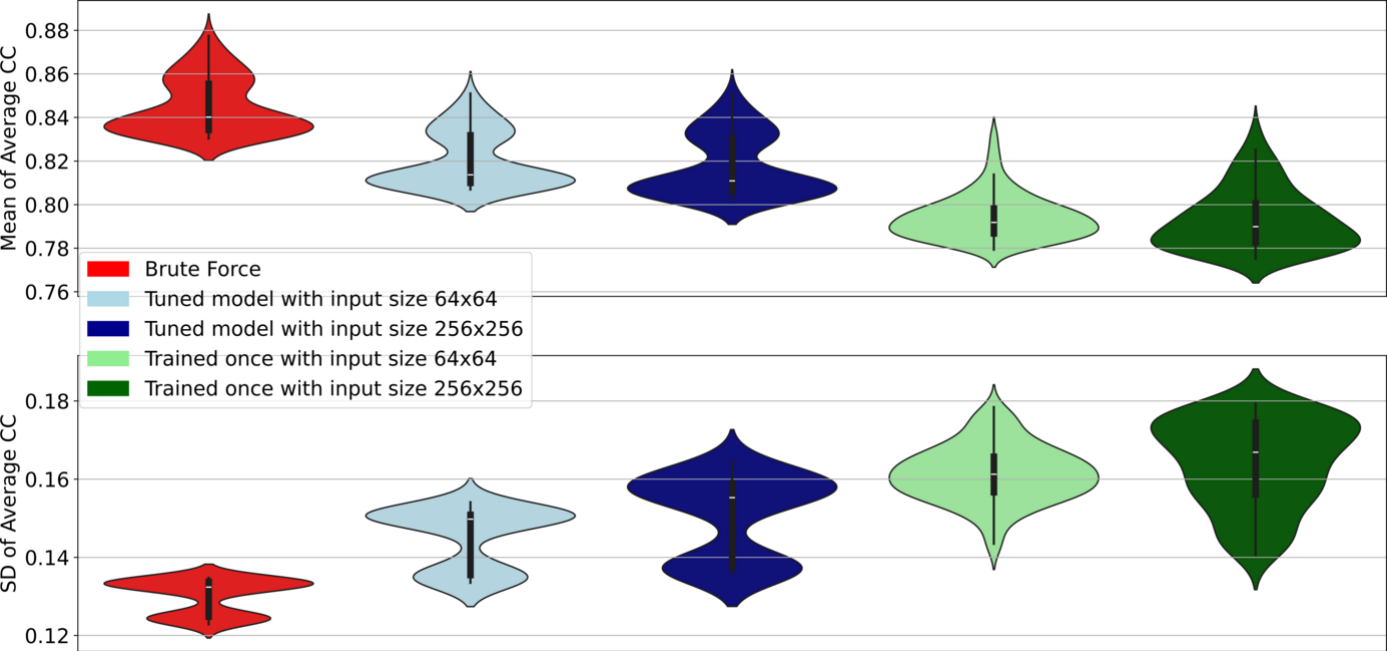
Variation of the average correlation coefficient (CC) per neighbor rank of the RSoXS images (first 100 neighbors for each image) using two fine-tuned models with inpainted X-ray scattering images of sizes 64 × 64 and 256 × 256, and a model trained from scratch without the inpainted images of sizes 64 × 64 and 256 × 256. As reference, a brute-force similarity search was used as ground-truth information, where the neighbor rank was assigned according to the values of the CC.

To evaluate further the impact of using pre-trained models for similarity-based labeling with small training data sets, such as the one with 166 data points, we compared the query results of models trained from scratch versus fined-tuned models from the inpainted data set. For this purpose, we analyzed the similarity between the queried images returned by both models using the Pearson correlation coefficient as our quantitative metric. Fig. 11 ▸ summarizes the results of this analysis through the mean and standard deviation of the average correlation coefficient of the 2697 RSoXS images in full resolution (1026 × 1024 pixels) with respect to their closest 100 neighbors. To do this, we identified the 100 closest neighbors for each image in our data set of interest based on the cosine distance between feature vectors, which was inferred from both the fine-tuned model with inpainted X-ray scattering images and a model trained from scratch without the inpainted images. To estimate the similarity between neighbors, we calculated the Pearson correlation coefficient between each image in the data set and its corresponding *n*th neighbor for later computation of the average correlation coefficient across neighbor ranks. Fig. 11 ▸ displays the mean and standard deviation of this average correlation coefficient per rank to summarize the similarity-based query performance across the used algorithms. These results clearly demonstrate that exploiting pre-trained networks can enhance the quality of a similarity-based labeling process without the need to move the complete remote data set towards the computing location, presenting both a higher mean and a lower standard deviation in the plots. The distribution of the average correlation coefficient is similar for the fine-tuned models and the brute-force algorithm queries, whereas the models that were trained once present a different distribution.

**Figure 12:**
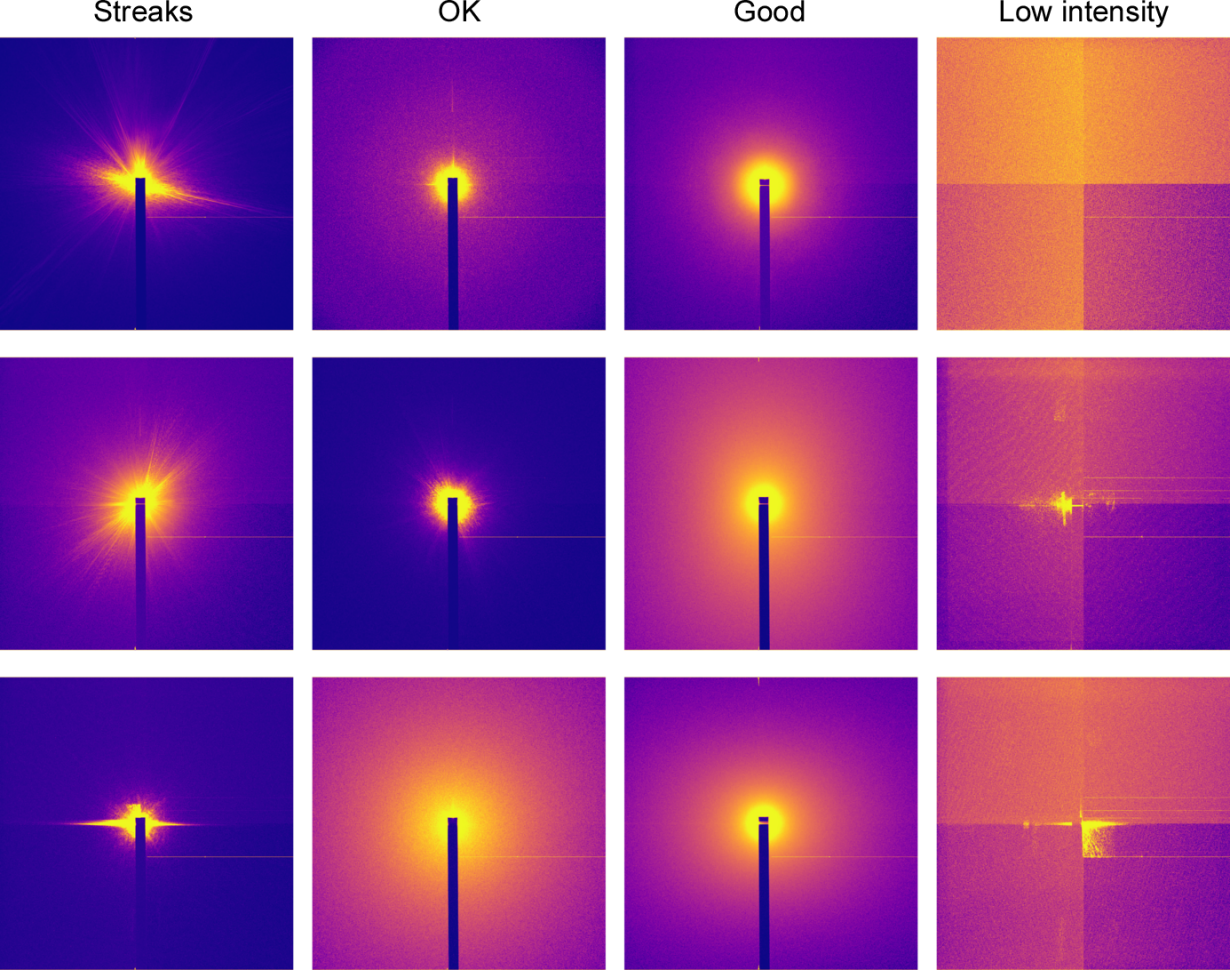
Sample images taken from the set of labeled RSoXS images with four categories, *Streaks*, *OK*, *Good* and *Low intensity*.

We labeled the RSoXS data set in *Label Maker* using the fine-tuned feature vectors through similarity-based batch labeling in addition to some manual labeling. The labels of interest within this data set were identified as *Good*, *OK*, *Streaks* and *Low intensity*, as shown in Fig. 12 ▸, with the patterns labeled *OK* presenting minor artifacts in the vicinity of the beam. At the end of the labeling process, 322 images were identified as *Low intensity* and 364 as *OK*, 1735 presented *Streaks*, and the remaining 157 were tagged as *Good*. The final results were stored in a database and downloaded to a filesystem. The labeling process time may vary according to the location and the internet speed between the *Tiled* server and the client browser, due to a potential waiting time increase in the data retrieval process.

### 4.3. Enabling fine-tuning of foundation models with human feedback

Providing high-quality labeled training data is crucial to enhancing the ability of foundational models to learn complex tasks, such as generating realistic X-ray scattering images. Thanks to its visualization capability, human interaction interface and integration with ML algorithms, the image labeling pipeline presented here can greatly facilitate the training of foundational models that require a large number of well labeled scientific data sets.

Using a fine-tuned stable diffusion model (von Platen* et al.*, 2022 ▸), we set up a text-to-image framework to generate X-ray scattering images from text prompts (Zhao *et al.*, 2024 ▸). However, due to the model’s generative nature, a portion of the generated images are not realistic. This is commonly known as ‘hallucinations’ in generative models (Aithal *et al.*, 2024 ▸). To address this issue, we used human-labeled images to train deep neural networks to identify highly realistic generated images automatically. This process needs a huge amount of labeled data. With the help of *Label Maker*, we were able to label accurately approximately 2700 (out of 20000) generated images and use these annotations to train a variety of classifiers [*e.g.* ResNet (He *et al.*, 2016 ▸)] in *MLCoach* to make the generative pipeline more robust against the identification of non-realistic data sets, which were validated by domain experts. Ultimately, this process brings human feedback into training of the classifier and it can be done in an iterative fashion until the classification performance reaches the desired accuracy.

Further implementation details and results of this study have been presented by Zhao *et al.* (2024 ▸), demonstrating that the labeling pipeline is instrumental in the generation of labels to train a set of classifiers iteratively to detect realistic versus non-realistic data points. As reported in this study, the model presented high-quality generated images as proven by their Fréchet inception distances (Heusel *et al.*, 2018 ▸) of 0.96, 0.62 and −8 × 10^−8^ for peaks, rings and background, respectively (Zhao *et al.*, 2024 ▸).

## 5. Discussion

The *Label Maker* pipeline provides an intuitive web-based tool to accelerate the labeling process of large complex scientific data sets using AI-guided labeling techniques. With the integration of *Data Clinic* and *MLCoach*, *Label Maker* makes use of both supervised and self-supervised learning techniques to power probability-based label assignment and similarity-based queries, respectively. As shown in Section 4, *Label Maker* has obtained promising results for three different use cases.

While this labeling pipeline has been thoroughly tested with X-ray scattering data sets, it is also well suited to other two-dimensional imaging data sets. Its versatility relies in the training process of its unsupervised and supervised algorithms through web interfaces, enabling the pipeline to effectively ingest and process various image-based data sets such as microscopy data. To enhance its labeling capabilities further, current short-term development plans for *Label Maker* aim to expand its capabilities towards the ingestion of one- and three-dimensional data sets, support for multiple assigned labels per data point, and the upgrade of *MLExCompute*. On the other hand, long-term development aspects focus on simplifying hyperparameter tuning and the availability of pre-trained weights, mitigating labeling errors, and supporting near real-time analysis, as discussed below.

### 5.1. Hyperparameter tuning and pre-trained weights

To use the AI-guided labeling techniques within *Label Maker* successfully, the selection of suitable neural network architectures and training parameters represents a crucial step to enhance the model performance and its generalization capacity to unseen data points. Considering this, the model configuration panel within *Data Clinic* and *MLCoach* provides a set of initial default parameters that can be used as a starting point for the training process of the ML models.

We can further simplify the hyperparameter tuning process by using robust pre-trained weights, ideally trained from large data sets with similar characteristics to the data set of interest. Currently, the probabilistic classifiers in *MLCoach* provide pre-trained weights based on the *ImageNet* data set (Deng *et al.*, 2009 ▸), which serve as a starting point for the training of a given classifier while also reducing the number of labeled images required to obtain a satisfactory performance. Alternatively, users can employ large data sets to pre-train a model for fine-tuning purposes, similarly to Section 4.2.

Hence, we aim to extend the library of pre-trained models for both unsupervised and supervised learning using large high-quality scientific data sets, such as the one collected on beamline 7.3.3 (Hexemer *et al.*, 2010 ▸).

### 5.2. Preventing labeling errors

**Figure 13:**
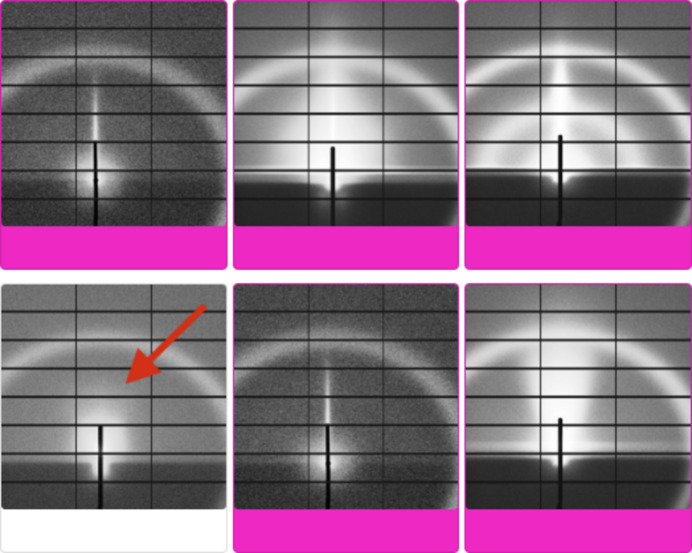
Example of potential labeling error with similarity-based batch labeling, where all the displayed images present a strong similarity with the exception of the area highlighted by the red arrow that presents a missing streak.

While *Label Maker* offers an assortment of AI-guided labeling techniques to accelerate the labeling process of complex data sets, errors can still occur during the label assignment process. For instance, Fig. 13 ▸ presents an example of a potential labeling error when using similarity-based labeling in *Label Maker*. Although these errors could be minimized through more robust trained models or training strategies, it is critical for *Label Maker* to offer alternative techniques for easy checking of the accuracy of assigned labels when data sets are large.

While alternative techniques like multiple instance learning (Maron & Lozano-Pérez, 1997 ▸) were initially excluded due to the complex nature of structural patterns in X-ray scattering that can overlap with or without direct correlation (Huang *et al.*, 2021 ▸), there is potential to incorporate them in the future for applications involving more heterogeneous data sets. The labeling pipeline can be further enhanced by incorporating advanced explainable AI methods, enabling scientists to build trust in the ML models and gain a deeper understanding of how the models arrived at the conclusions presented in the interface. Examples of such methods include occlusion maps (Zeiler & Fergus, 2014 ▸), class activation maps (Zhou *et al.*, 2016 ▸; Selvaraju *et al.*, 2017 ▸) and self-attention-based maps for vision transformers (Chefer *et al.*, 2021 ▸). Additional efforts in visualization and software architecture design are essential to integrate these tools seamlessly, ensuring they are accessible and user friendly.

Further development plans for *Label Maker* include the integration of both an active learning analysis pipeline and random validation checkpoints to mitigate labeling errors. With these proposed features, users will be able to receive validation checkpoints at different stages during their labeling session. At these checkpoints, users will be challenged with re-labeling a set of previously labeled images selected randomly or by a learning agent. According to the results of this checkpoint, users will verify that the same labeling criteria are applied throughout the entire data set and fix potential errors if needed.

### 5.3. Near real-time data analysis

Several capabilities that are offered by the *Label Maker* pipeline can have a positive impact for the analysis of complex data in near real time during experiments. In particular, autonomous frameworks with human involvement such as that presented by Biswas *et al.* (2024 ▸) could benefit from on-the-fly labeling capabilities, where users upvote or downvote spectral data to steer experiments. Therefore, there is potential interest in the expansion of this labeling pipeline to ingest and analyze experimental data in near real time.

The integration of *Latent Space Explorer* to this labeling pipeline will enable users to better visualize the latent space obtained from the tunable autoencoders, which undergoes a dimension reduction step prior to visualization. Hence, users will be able to make informed decisions based on visual inspection of the latent space and tune parameters accordingly in near real time.

## 6. Conclusions

This paper has introduced an ML-based labeling pipeline for scientific data sets that offers both manual and AI-guided labeling approaches through unsupervised and supervised ML models. This labeling pipeline combines three web-based applications, *Label Maker*, *Data Clinic* and *MLCoach*, to tailor the selected ML models for their particular use case prior to labeling. The experimental results have presented three use cases emphasizing the labeling capabilities of *Label Maker* to tag large scientific data sets, to fine-tune foundation models with human feedback and to analyze RSoXs data sets remotely.

Through these use cases, we have gained valuable insights into *Label Maker*’s potential impact in various scientific fields, such as materials discovery. For instance, *Label Maker* has enabled the creation of ground-truth tags for historical X-ray scattering data sets collected at the ALS over the past decade for the detection of structural properties, as shown in Section 4.1. The integration of *Tiled* within this labeling pipeline has enabled the remote analysis of RSoXS data with fine-tuned autoencoders by using inpainted X-ray scattering images as presented in Section 4.2. *Label Maker* and *MLCoach* were instrumental for the preparation of image discriminators to enhance the robustness of generative models, such as the one presented in Section 4.3.

These results demonstrate the promising capabilities that *Label Maker* enables for the ML-based analysis of scientific data sets. This labeling pipeline lowers the introduction barrier to ML techniques, since software development experience is not required to deploy training and inference processes within its web user interfaces. With the further integration of *Latent Space Explorer* and an extensive library of pre-trained weights, we aim to reduce further the complexity of tuning hyper­parameters to boost the performance of ML approaches.

## Figures and Tables

**Table 1:** Uniform resource identifiers of labeled RSoXS *Bluesky* runs, where the cells highlighted in **bold** represent the small subset that was used to train and fine-tune the autoencoders that were used for similarity-based labeling

**0612cb98-29c6-49e2-9a1a-780f7b9f2365**	06e1511a-2470-4176-a484-59aadecdf09b
077c21aa-66ec-4ec3-84bc-1dcfaa590751	08add695-5558-4276-95bd-3cd278e8a63c
0f75fa7f-9cc0-4b21-9eb3-93b661144186	117b9502-b82a-4a6d-880c-7b69c746aecd
196d936b-9bdc-4d5e-a27b-43c4022ddef1	24f6eca4-d5c5-44e6-b7d5-188941dfd271
2bf14fec-5789-4edd-bb92-0aa48ea2bb62	3044480e-57d3-466b-b25f-d4df88c2bb68
3e8f80e3-e78b-4df3-945f-646e6fe9ae5f	3f5851a5-3e0f-4453-8b65-d4bdd899fcc4
402602f8-8d7e-4772-b1c6-0fd03c2d2870	41baefa5-71a4-4171-b420-475c1e16f1e8
4232bdd2-485f-4880-baaf-700710e67863	42390e5e-1ebd-4671-9610-83cf8e6ccc4c
43733bfe-a2c2-4c94-abde-5574ff75f2f8	468810ed-2ff9-4e92-8ca9-dcb376d01a56
49f11cbe-77bb-431e-bdfc-126351ee533e	4a6090de-2bec-4cce-b9b7-77c962336da3
4a8e6d20-09f8-4d4b-9eee-1b8a705ff9dc	4b619f82-8109-494c-aa80-2016fdae9162
4d22a49c-6a6e-4300-9723-242a7624c411	4d73c2f0-0853-44f3-a457-a815f7c671a2
4e1e2ad6-0bfe-4b86-aa90-a8506a9821c1	4e4b11a0-f9e6-4d87-be09-c2e67eb31373
4fa0282a-f3e9-42eb-bcca-353c9b97bf2f	521a0d85-3dbb-49d7-b790-273dffa01adf
575e38bc-b436-4574-8998-271abbe8789b	5be6565a-22c2-4fe3-92f0-29e6da75be17
**5c15445c-cfd2-43f9-a7b1-e22588a62218**	5e57263b-395f-4c26-86ae-938a59cf76bd
5eb289fe-1405-489d-b60d-254bfe80fdfa	**62a39bd1-6fd0-40f9-98d4-35578c643929**
64d2d687-efd3-4b78-87f8-b105a5941356	66194d57-3394-48a6-af81-c6a571e9253d
6d65e6a5-6f68-4fda-b0e2-c355da3a2298	70c3fc88-b1e7-4dc4-a1bd-6694805e187e
7259fba5-efdc-48fd-b6cc-0315f5737187	77b7cc7b-9011-4caa-a9d5-d23f1d207ea6
78c4becf-b0c6-4385-8d65-ba5a7d801a40	7ed73976-a074-40d6-9a31-c443e6f8af22
7ef20e0a-8fe3-4091-8bfd-bf28ce4b1c4c	80ec2d8d-9936-4438-8b5d-c59bed520832
89acb459-0630-4bce-bf58-c2ddd54fbdfb	**8a25ab24-6a77-43bc-99ab-e75cc3437b14**
8a5a6cb4-ed73-42d5-86b6-082327 dd649a	91597d7e-cbe8-4920-92f4-12fc105363ec
92816b30-abe0-400d-8fca-71ac53e8fe0d	92ed98a5-ba7e-45cb-9ac2-ca4613618f2d
9549a5ff-8c18-458e-b51b-32df89dc1a89	9e1ef5a7-80d8-4c99-9b26-189af6cafa5a
a6a21ac2-e5d1-4fdf-9ce5-70fd04c57359	a70c2e3e-a015-48b6-89bb-01787bd429d7
acc7408b-daab-4516-9c2c-56f7ea179512	b37dd4e6-c5ea-45bc-8a04-2ad3da6a1167
b423ff0a-260c-4251-adfb-ddec78c5ee15	b483328f-1011-4a67-9e60-9d292b49a470
b7b4740f-17a4-4827-ad23-d5f70fa4a8e4	b8550b08-233d-4f18-9b36-bae8f991e7de
ba83ab2d-7722-4dff-abb4-80e13b30b0c6	be40580f-651e-4f41-ae0b-cbb61be8aeec
c44368f9-ebb2-4b49-935e-cd5e1c01ad7b	c5c44bb2-1737-469f-813e-0b5cebf21911
c65de975-2ca7-4036-a3fd-58dd593f4b9f	c9db2c01-83aa-4aa6-a1c2-e04722deef22
ccb66c36-6ee2-4107-8e17-ce6f54cca115	d2abb596-a76d-4b59-99a4-c9ae08279114
d491b68e-f829-4d8b-975d-648dd51cbc97	dae99200-fb38-40c5-b1a0-b74f1ceaea71
dd1ec7e6-e53c-4d1f-8205-7f0396571836	de9e6427-7c20-43e3-bcd6-840baf7ee51c
e0d82ec1-96dd-4cce-a91b-cdead0e92f1f	e11fad16-1b59-426f-ba07-1de6fe04c3b2
e36689d2-db97-45e5-a39b-9137ae1faadf	eb6c0565-d671-4e05-959e-e045af9ff062
ef6d575f-677a-4450-ac62-a320422991fa	f00acbef-9c7a-430b-92f7-bc1abdd576d0
f3bfe133-9613-49ab-a3e1-4a4f91944b3f	f78c94aa-eba5-4c30-a8ba-2dfc6dcda1e0
fa51969c-c7d6-4aa3-9c49-5ce82b2e99fb	fac2923d-1519-4491-a3fb-66885e10ee97
fb09ee25-f95f-4712-8b3b-0833dd21c268	fcd40ef0-1bc6-4e46-8b65-2222c3d89296
**fe0b6a01-3f49-4fdc-847f-dca4c91cd36b**	

## Appendix A Data availability

The X-ray scattering data set used in this study, collected on the SAXS/WAXS beamline 7.3.3 (Hexemer *et al.*, 2010 ▸), is available through Globus at https://app.globus.org/file-manager/collections/e393df21-17aa-4c2d-8739-7bf80cb4f26b/overview.

The resonant soft X-ray scattering (RSoXS) data set used in this article is publicly available at https://tiled-demo.blueskyproject.io/ui/browse/rsoxs/raw. The selected *Bluesky* runs that were used for this study are presented in Table 1 ▸.

## Appendix B Code availability

*Label Maker* is an open source project. The source code for the various components can be found at:

(i) *Label Maker*: https://github.com/mlexchange/mlex_labelmaker.

(ii) *Data Clinic*: https://github.com/mlexchange/mlex_data_clinic.

(iii) *MLCoach*: https://github.com/mlexchange/mlex_mlcoach.

(iv) *File Manager*: https://github.com/mlexchange/mlex_file_manager.

(v) Tunable autoencoders: https://github.com/mlexchange/mlex_pytorch_autoencoders.

(vi) Image classifiers: https://github.com/mlexchange/mlex_image_classification.

(vii) *Splash ML*: https://github.com/als-computing/splash-ml.

(viii) *MLExchange*: https://github.com/mlexchange/mlex.

## Appendix C Hyperparameter selection

Most of the hyperparameters used for this study were chosen from the default suggested parameters in the web applications with very minimal changes. In particular, the training parameter selection for the analysis of RSoXS data was the result of a hyperparameter sweep, where we analyzed the impact of changing the input image size as 64, 128, 256 and 512, and selecting a latent vector dimension of 300 and 900. The network architecture for each combination was adjusted accordingly. The results of this sweep demonstrated that changes in the image size were minor for the tuned model with inpainted scattering data, whereas the model that was trained once presented a slightly higher distribution in its standard deviation as shown in Fig. 11 ▸. Note that the changes in latent vector dimension had minimal impact in both models. Hence, this study summarizes the corresponding findings for the smaller size, corresponding to 300.
